# Online dietary intake assessment using a graphical food frequency app (eNutri): Usability metrics from the EatWellUK study

**DOI:** 10.1371/journal.pone.0202006

**Published:** 2018-08-10

**Authors:** Rodrigo Zenun Franco, Rosalind Fallaize, Julie A. Lovegrove, Faustina Hwang

**Affiliations:** 1 Biomedical Engineering Section, School of Biological Sciences, University of Reading, Reading, United Kingdom; 2 Hugh Sinclair Unit of Human Nutrition and Institute for Cardiovascular and Metabolic Research, University of Reading, Reading, United Kingdom; McMaster University, CANADA

## Abstract

**Background:**

With widespread use of the internet, lifestyle and dietary data collection can now be facilitated using online questionnaires as opposed to paper versions. We have developed a graphical food frequency assessment app (eNutri), which is able to assess dietary intake using a validated food frequency questionnaire (FFQ) and provide personalised nutrition advice. FFQ user acceptance and evaluation have not been investigated extensively and only a few studies involving user acceptance of nutrition assessment and advice apps by older adults are published.

**Methods:**

A formative study with 20 participants (including n = 10 ≥60 years) assessed the suitability of this app for adults and investigated improvements to its usability. The outcomes of this formative study were applied to the final version of the application, which was deployed in an online study (EatWellUK) with 324 participants (including n = 53 ≥60 years) in the UK, using different devices (smartphones, tablets and laptops/desktops). Completion times were based on browser timestamps and usability was measured using the System Usability Scale (SUS), scoring between 0 and 100. Products with a SUS score higher than 70 are considered to be good.

**Results:**

In the EatWellUK study, SUS score median (n = 322) was 77.5 (IQR 15.0). Out of the 322 SUS questionnaire completions, 321 device screen sizes were detected by the app. Grouped by device screen size, small (n = 92), medium (n = 38) and large (n = 191) screens received median SUS scores of 77.5 (IQR 15.0), 75.0 (IQR 19.4) and 77.5 (IQR 16.25), respectively. The median SUS scores from younger (n = 268) and older participants (n = 53) were the same. The FFQ contained 157 food items, and the mean completion time was 13.1 minutes (95% CI 12.6–13.7 minutes). Small, medium and large screen devices resulted in completion times of 11.7 minutes (95% CI 10.9–12.6 minutes), 14.4 minutes (95% CI 12.9–15.9 minutes) and 13.6 minutes (95% CI 12.8–14.3 minutes), respectively.

**Conclusions:**

The overall median SUS score of 77.5 and overall mean completion time of 13.3 minutes indicate good overall usability, and equally, comparable SUS scores and completion times across small, medium and large screen sizes indicates good usability across devices. This work is a step toward the promotion of wider uptake of online apps that can provide online dietary intake assessment at-scale, with the aim of addressing pressing epidemiological challenges.

## Introduction

Non-communicable diseases such as diabetes and cardiovascular diseases account for almost two thirds of deaths globally. The general recommendations for addressing this epidemic are related to lifestyle changes, mainly encouraging healthy diets, physical activity (PA) and the reduction of tobacco use and alcohol consumption [1]. It is also estimated that 3 million people in the UK are malnourished or at risk of malnutrition, and of those, a third are over the age of 65 and 93% live in the community [2].

Due to the widespread use of the internet, lifestyle and dietary data collection can now be facilitated by use of online questionnaires as opposed to paper versions due to the widespread use of the internet [3]. Food Frequency Questionnaires (FFQ), which are used for food and nutrient intake analysis, are an example of such a data collection method [4]. We have developed a graphical food frequency assessment app, which is able to assess dietary intake using a validated FFQ [5] and provide personalised nutrition advice. Users of this app record frequencies of food items consumed during the last month (e.g. "1/week" or "1/day") and select one of three portion size images for each specific food item [6].

This online app (named eNutri) aims to encourage healthier eating. In order to achieve this goal, it is essential that it has good user acceptability by the target adult population, including older groups. FFQ user acceptance and evaluation have not been investigated extensively and there are only a limited number of studies involving user acceptance of nutrition assessment tools by older adults [7–8]. Age-related changes in cognitive, perceptual and motor capabilities affect how older people interact with technology [9], and particularly for older adults, it is important to consider particular design principles, such as limiting the number of on-screen choices available to the user, using appropriate font size and avoiding hidden items [10].

Some studies using web-based graphical FFQs have reported data for user acceptance, using tailor made usability questionnaires (i.e. non-standard) [5,11–12]. These approaches for assessing usability and the lack of public access to the raw data greatly increases the challenge of employing users’ feedback to improve similar tools. The interfaces of these web-based FFQs [13–14], were not designed to be used on smartphones (i.e. non-responsive web design). Furthermore publications on these FFQs focus on the validity and reproducibility of the method from a nutritional perspective [13,15]. A recent publication presented a dietary assessment tool consisting of a 24-hour dietary recall (24HDR) and a FFQ which seems to be responsive, although it was not explicitly stated that this tool could be used on smartphones (i.e. small screen devices) [16] and the source code was not available as open source. FFQ completion times by device types were reported for the first time in this article.

This study evaluated the suitability of this app for adults and investigated improvements with its usability. The description of the design decisions, their use in the app and related feedback from the study participants are important contributions to the research community interested in deploying online apps for nutrition assessment. These insights could also be applied in similar apps, especially in the digital health domain.

## Materials and methods

### eNutri application

#### Physical activity questionnaire

The physical activity level was assessed via the Baecke Questionnaire [17], which is a short questionnaire for the measurement of habitual physical activity. It has been validated and found to be repeatable [18] and it has been used in studies similar to this one [19].

#### Food frequency questionnaire

Our application was developed independently (i.e. original source code), however it employed the food list and related portion size images from an existing FFQ (Food4Me) that was previously validated in the UK [5]. The web design version used in the formative study was published in [6]. Each food item is presented individually on the screen, such that the user has only one navigation path through the FFQ. This design was also motivated by evidence showing that a linear style is preferred by older adults [20].

#### Usability metrics

Just after completion of the FFQ, the app presented a System Usability Scale (SUS) [21] questionnaire. This standard usability metric contained 10-items that relate to a range of aspects of app use, such as complexity, ease-of-use, and learnability (e.g. “I thought the system was easy to use."), each with 5 response options from “Strongly disagree” to “Strongly agree”. Products with a SUS score higher than 70 are considered to be good [22]. After the 10 items, the overall usability of the app was evaluated via a general question "Overall, I would rate the user-friendliness of this system as:" with the following options: "Worst Imaginable", "Awful", "Poor", "Fair", "Good", "Excellent", and "Best Imaginable". The last usability question collected textual feedback via the question: “Have you had any difficulties with using the system?”

Timestamps on actions completed during the completion of the FFQ (i.e. clicks to move to the next food item) and browser details (e.g. device screen size) were automatically logged by the app, using the JavaScript Date Object [23] and the Navigator and Screen interfaces [24]. The timestamps were analysed for the total time spent completing the FFQ. The device screen sizes were classified as small (less than 480 pixels wide), medium (between 480 and 1240 pixels wide, inclusive) and large (more than 1240 pixels wide). In touchscreen devices, this application was used in the portrait position only, hence this classification can be interpreted broadly as handsets (smartphones), tablets, and laptops/desktops [25].

### Formative study

#### Participants

The ultimate aim was that the eNutri app would be entirely self-administered by adults. To this end, it was important to assess whether target users were able to complete the user journey without assistance. In Human Computer Interaction studies, a small number of participants can effectively detect errors and highlight necessary improvements to a system [26].

In this formative study, adult participants (18+) were recruited from the Hugh Sinclair Unit of Human Nutrition, University of Reading volunteers’ database via e-mail, they were stratified into two groups based on age (18–59 and 60+ years). The recruitment and the study occurred between April and May 2017. Participants received a £5 shopping voucher for their participation.

#### Procedure

After a participant gave written informed consent, demographic characteristics (age, sex, height, weight, level of education) and familiarity with technology were assessed via a paper-based questionnaire at the beginning of the study. The level of familiarity of the participants with technology was assessed via four questions regarding the frequency of use of computer devices, device ownership, Internet use and main device used to access the Internet. In the first question, participants were asked to report how often they use common computer devices. At the beginning of the experiment, it was explained that participants should try to complete the process without asking for help. Notes were taken regarding the point of stopping and related difficulties if the participant was unable to proceed. A researcher created an account in the app for the participant, using an iPad 4^th^ generation (9.7-inch Retina display, 768 x 1024 pixels) running Google Chrome. The device was then handed to the participant for study commencement.

The app then asked participants to complete the Baecke Questionnaire for collecting basic physical activity information, the FFQ and the SUS questionnaire before proceeding to the semi-structured interview, which was designed to collect qualitative data relating to usability challenges with the app that could not be captured using the SUS or via the online forms. The first section of the interview focused on the FFQ (nutrition assessment) with questions regarding the participants’ experience of using the app, what they liked and which aspects could be improved. The second section of the interview explored participants’ understanding of the online report, which will be submitted for publication in another article.

A total of 20 participants (with n = 10 ≥60 years) were recruited. Their demographic characteristics and technology familiarity (see Table A, Table B and Table C in S1 Table) and the raw data collected are available in the S1 Table.

#### Results

All participants were able to complete the Baecke questionnaire without any difficulties, although four participants who were retired found the questions on work activities inappropriate. Five participants mentioned that either the division of sports and leisure categories were confusing or the examples [17] did not reflect the most common activities in the UK.

All participants were able to complete the FFQ without assistance from the researcher. None of the participants clicked on the main help button, which was visible at the top right of the screen [6]. Although it was not part of the original study protocol, during the interview, the researcher also asked 10 participants if they had noticed the progress indicator displayed in the FFQ screens [6]. Three declared that they had not noticed it during the FFQ completion.

During the semi-structured interview, 16 participants mentioned the main advantage of the app was its “easy-to-use” aspect, using this specific term (n = 12) or related terms such as “simple” (n = 2), “intuitive” (n = 1) or “friendly” (n = 1). The other 4 participants mentioned that they “enjoyed it”, found the app “quite interesting” and “well setup” (Table G in S1 Table).

Six participants did not report any areas for improvement of the app. Eleven participants mentioned that the frequency selection could be improved, of which 5 stated that the order of the options [6] was not intuitive or made the decision-making process more demanding. Suggestions such as decreasing the distance between the columns or replacing the “/” with “per” and “>” with “more than” were also mentioned. Five participants stated that the portion size images could also be improved, because some of portion size options were very similar to each other or not presented as they were expecting (e.g. presenting different portions of bread as varying bread slice sizes instead of increasing numbers of slices of the same size; displaying beer bottles instead of pints).

The mean SUS score for the whole group (n = 20) was 76.9 (IQR 13.1), for the older group (n = 10) was 72.8 (IQR 10.6) and for the younger (n = 10) was 81.0 (IQR 13.8) (S1 Fig). All participants completed the FFQ without interruption. The mean completion time (n = 20) was 22.9 minutes (95% CI 19.7–26.1 minutes), with a range from 10.5 to 39.0 minutes. The mean for the younger group was 19.4 minutes (95% CI 16.2–22.6 minutes) and for the older group was 26.4 minutes (95% CI 21.2–31.5 minutes) (S2 Fig). The FFQ contained 157 food items, and the mean time per food item was 8.7 seconds.

**Fig 1:**
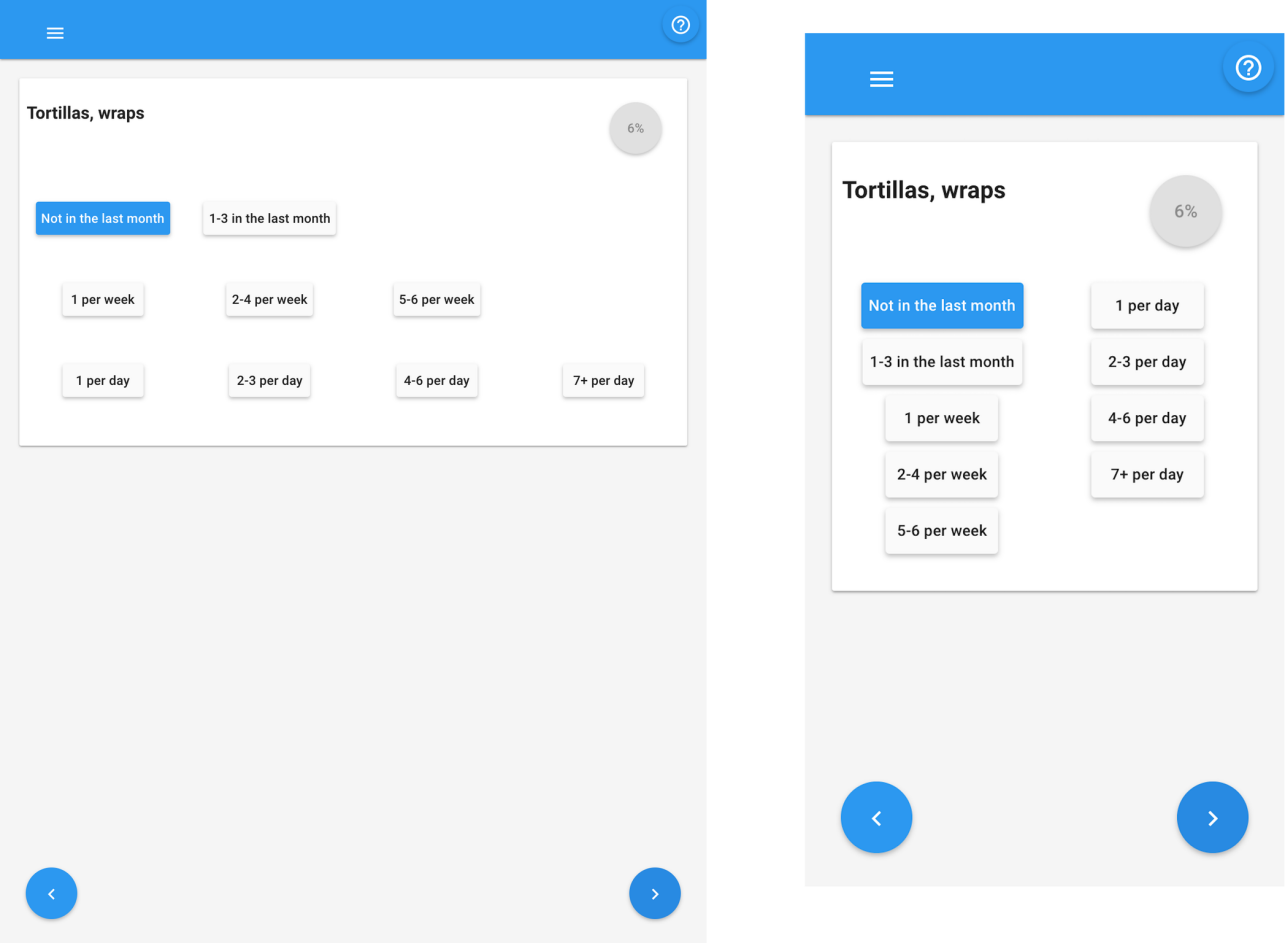
Food frequency options reordered and presented in medium and small screens.

The main suggestions and related improvements applied to the online FFQ were:

- Move automatically to the next food item after selecting “Never” or the portion size, without requirement to click the forward arrow;
- Reorder the buttons for selecting frequency of consumption to better facilitate selection (grouped by month, week and day). The layout was modified so that it would adapt to the device screen size (i.e. present the buttons in two-columns for small screens and in four-columns for medium and large screens). This modification can be seen by comparing the previous version of the eNutri app [6] and Fig 1;
- The frequency “Never” was modified to “Not in the last month” to clarify that, in completing the questionnaire, participants should only report what they consumed in the last month, rather than estimating their average consumption of that item over a year.

These modifications were applied before the start of the EatWellUK study, which took advantage of the suggestions provided by the participants in the formative study.

### EatWellUK study

#### Participants

Participants resident in the UK were recruited via e-mail, social media and online advertisements, between August and November 2017. This study recruited a generally “healthy” adult population, that is, adults of all ages who had no diagnosed health conditions. The following pre-requisites were applied during recruitment and screening: Adults without a diagnosed disease condition (e.g. diabetes, heart disease); not pregnant nor lactating; no food allergy nor intolerance; not on a specific diet (e.g. vegan); not receiving face-to-face nutritional services (e.g. from a nutritionist or dietitian) and able to speak English fluently. The participants would not receive money for their participation.

#### Procedure

During the EatWellUK study, potential participants were requested to go directly to the study website, create an account, give consent and confirm their eligibility criteria. Similar to the formative study, they were also asked to complete the Baecke, FFQ and SUS questionnaires. Participants were encouraged to complete the FFQ in one session, but informed that if they had to leave the computer, responses would be saved and valid for 24 hours. As the browsers’ timestamps were absolute values [23], intervals greater than 60 seconds between food items were considered breaks and replaced with an estimated completion time per food item (i.e. 10 seconds).

The formative and EatWellUK studies were subject to ethical review according to the procedures specified by the University of Reading and conformed with the Declaration of Helsinki. The School of Chemistry, Food and Pharmacy Research Ethics Committee approved these studies (Ref No. 04/17 and 13/17, respectively). The EatWellUK study was registered in the ClinicalTrials.gov (NCT03250858).

## Results

### EatWellUK study

#### Participants

A total of 439 participants created an account on the study website and 365 were accepted after screening. Of the 365 enrolled onto the study, 324 completed the baseline FFQ. The demographic characteristics of these 324 participants are shown in Table 1.

**Table 1:** Demographic characteristics of the participants (n = 324) who completed the EatWellUK baseline FFQ.

Characteristics	Total	%
**Sex**		
Female	258	79.6
Male	66	20.4
**Level of Education**		
Less than secondary	1	0.3
Secondary	43	13.3
College	39	12.0
Bachelor	115	35.5
Postgraduate	126	38.9
**Age group**		
Younger (<60)	271	83.6
Older (> = 60)	53	16.4
**Age (years)**		
Mean	42.16	
Range	18–85	
**BMI (kg/m**^**2**^**)**		
Mean	25.1	
Range	16.5–60.8	

#### Usability metrics

**Fig 2:**
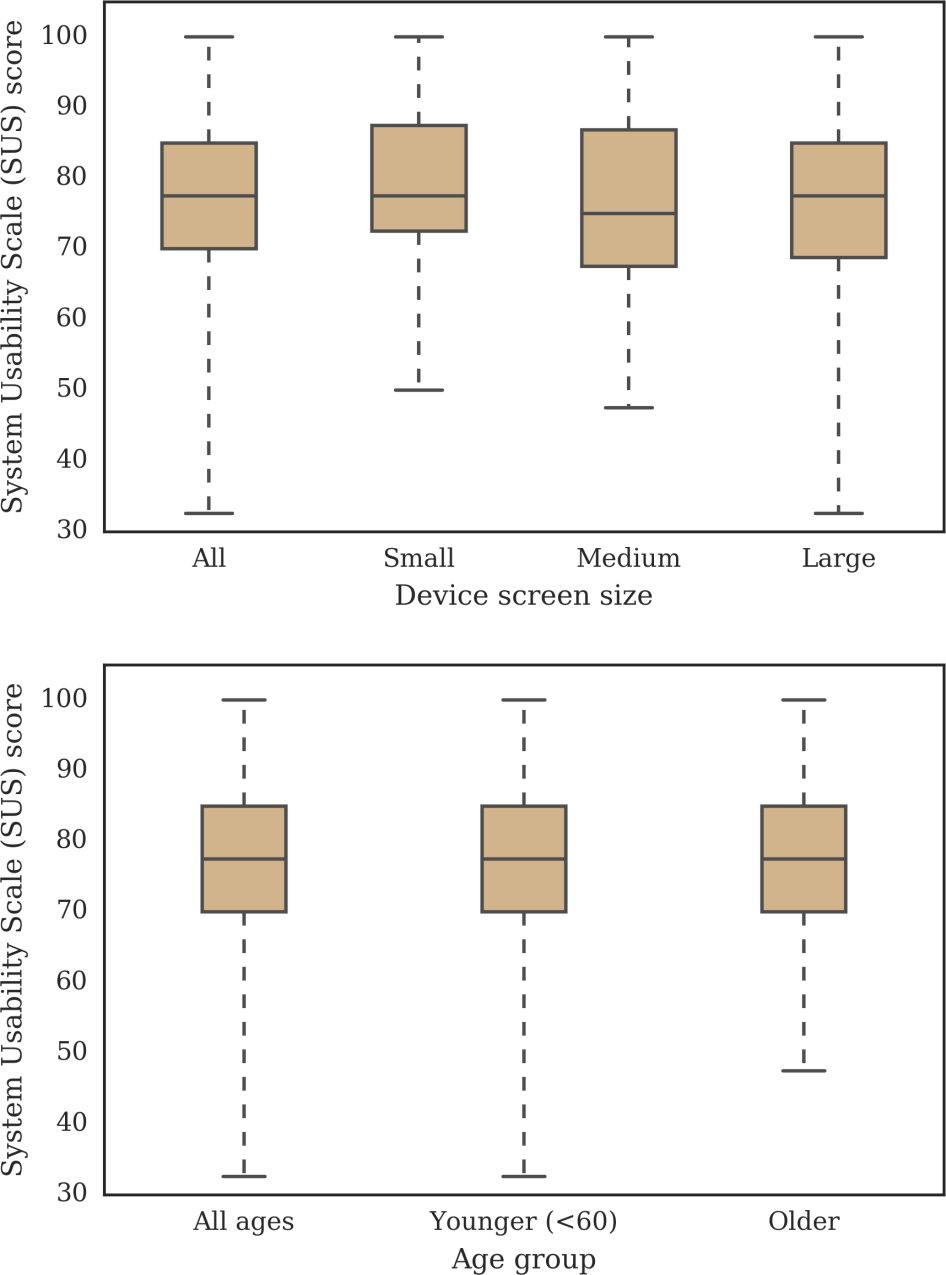
System Usability Scale (SUS) score for participants (n = 322) who completed the EatWellUK study presented by small (n = 92), medium (n = 38) and large (n = 191) device screen sizes and by younger (n = 268 <60 years) and older (n = 53) adults. The device screen sizes were classified as small (less than 480 pixels), medium (between 480 and 1240 pixels, inclusive) and large (more than 1240 pixels).

Two participants completed the FFQ but did not complete the SUS questionnaire. The median SUS score (n = 322) was 77.5 (IQR 15.0). Out of the 322 SUS questionnaire completions, 321 device screen sizes were detected by the app. Divided by device screen size, small (n = 92), medium (n = 38) and large (n = 191) screens received median SUS scores of 77.5 (IQR 15.0), 75.0 (IQR 19.4) and 77.5 (IQR 16.25), respectively. The median SUS scores from younger (n = 268) and older (n = 53) participants were the same at 77.5 (IQR 15.0) (Fig 2). The aim here was not to compare for statistical differences between age groups nor screen size, only to gain further insight about how the data are distributed in order to know if there were any particular difficulties experienced by subsets of the sample. The results suggest comparable performance across age groups and screen sizes.

The overall perceived quality of the app was reported as either “good” or “excellent” for 81% of the 322 participants (Table 2).

**Table 2:** Overall perceived quality of the eNutri app by participants (n = 322) of the EatWellUK study presented by small (n = 92), medium (n = 38) and large (n = 191) device screen sizes.

	All devices	Small screen	Medium screen	Large screen
Perceived quality	%	%	%	%
Best Imaginable	4.7	8.7	7.9	2.1
Excellent	39.4	45.7	13.2	41.4
Good	41.6	38.0	60.5	39.8
Fair	13.0	7.6	18.4	14.7
Poor	0.9	0.0	0.0	1.6
Awful	0.3	0.0	0.0	0.5
Worst Imaginable	0.0	0.0	0.0	0.0

**Fig 3:**
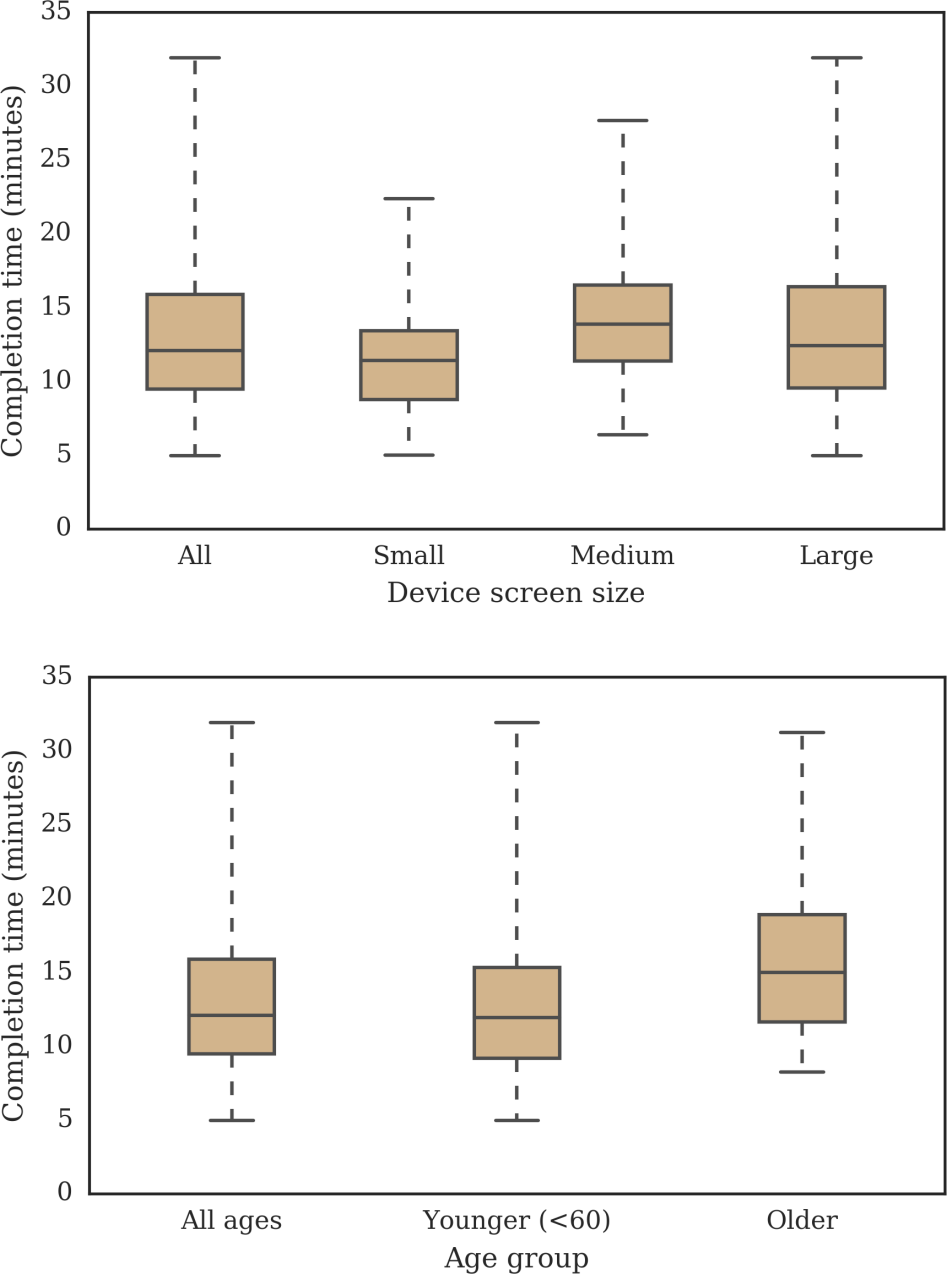
FFQ completion time for participants (n = 324) during the EatWellUK study presented by small (n = 92), medium (n = 38) and large (n = 191) device screen sizes and by younger (n = 271 <60 years) and older (n = 53) adults. The device screen sizes were classified as small (less than 480 pixels), medium (between 480 and 1240 pixels, inclusive) and large (more than 1240 pixels).

The mean FFQ completion time was 13.1 minutes (95% CI 12.6–13.7 minutes); for small, medium and large screen devices was 11.7 minutes (95% CI 10.9–12.6 minutes), 14.4 minutes (95% CI 12.9–15.9 minutes) and 13.6 minutes (95% CI 12.8–14.3 minutes), respectively. The younger adults (n = 271) completed the FFQ in 12.6 minutes (95% CI 12.1–13.2 minutes) and the older adults (n = 53) in 15.8 minutes (95% CI 14.3–17.3 minutes) (Fig 3). The aim was not to statistically compare the groups but to gain further insight into how completion times were distributed across groups, and the results suggest comparable completion times across age groups and screen sizes.

In the final question (“Have you had any difficulties with using the system?”), 45 participants (n = 322) answered “yes”. Further examination of the comments revealed that 10 were blank or included clarification that they had not experienced any problems. Six participants mentioned that the Baecke questionnaire was not suitable for retired participants and another six commented on the portion size images. Nine comments suggested issues with browser compatibility or Internet connection.

## Discussion

This study presents the design of a graphical FFQ providing online personalised nutrition advice. The usability and user acceptance are presented initially in a beta version of the application, which was used in a formative study in order to validate its suitability for younger and older adults. Insights from the formative study were detailed and applied to the new version of the application, which was used in the EatWellUK study including 324 participants.

The completion of all the questionnaires without assistance and a good overall SUS score of 76.9 were important in confirming the suitability of this application for an online study including older adults. The mean completion time of 22.9 minutes (IQR 6.6) suggested that some improvements might be necessary in order to facilitate participants’ completion of the questionnaires. Key points for improvement highlighted by the formative study included an opportunity to have the app automatically moving to the next food item after a portion size had been selected and reordering of the food frequency options to make it easier for users. These two options impact the FFQ completion time, by reducing the number of clicks per food item and the decision-making time. Especially for very repetitive tasks, such as the FFQ, such modifications can impact drastically the completion time. The reduction from 8.70 to 5.02 seconds per food items in the completion rate indicated that these changes were effective.

The modification of the frequency “Never” to “Not in the last month” was an example of how simple and important modifications can emerge from formative studies. Kurtom and Bangor measured popular services and products and reported a SUS average of 70.14. SUS scores of popular applications, such as Microsoft Excel (56.5), GPS (70.8), and an automated teller machine (82.3) can be used as references [27]. This version of the application presented the food items individually on the page and received a median SUS score of 77.5 (IQR 15.0), which is slightly higher than our previous version of the application (75.0 (IQR 12.5)), which presented all the food items as one large list on the screen [28]. The myfood24 project reported their SUS results of an online 24HDR system and for an adult population, it resulted in a median of 68 (IQR 40) for the beta version, and a median of 80 (IQR 25) for the final version [29]. No similar results have been published for online FFQs, especially presenting the data by device type.

This new completion rate (i.e. 5.02 s/item) was faster than the previous one using a food list (5.84s/item) [28]. An online FFQ deployed in Spain reported a completion time of 15 minutes for 84 food items (14). This represents 10.34 seconds per food item, which is more than double the completion rate for eNutri app, although it is not clear if their completion time measure only the FFQ completion.

Although the results from these separate studies are not directly comparable because they were conducted in two different contexts and populations, these results do give an indication that the new design has improved the eNutri app, and that the design features offered a good level of usability and user acceptance. Considering there are one to two decisions per food item, this completion rate seems to indicate a good flow in the process. Further drastic reductions in this completion time are likely to be challenging, suggesting that other alternatives should be explored if the completion time is still not acceptable for a specific use. An alternative could be to reorder and reduce the food list dynamically, based on the participant’s previous responses, via recommender systems techniques.

One of the challenges of designing online graphical FFQs is the ability to deploy the application on different devices, especially with the limited space on smartphone screens. This was one of the main motivations for examining the data by device screen sizes. The comparison of the SUS scores (Fig 2) and completion times (Fig 3) by screen size indicate the suitability of this web application for any device. This FFQ presented three portion size images per food item, making it possible to present them on-screen simultaneously, even on small screen devices. A need to increase the number of portion size images would demand a change in the design and potentially impact the completion time if additional clicks were added to the process. Other web-based online FFQs may not be suitable for smartphones due to the amount of information on-screen and would require a new responsive design [4,12,15].

The number of older adults (n = 53) was smaller than younger adults (n = 271), however the results of the SUS score and completion time for the older group indicated good suitability of this application for this population. The authors of this paper are not aware of a similar online nutrition assessment usability study including this number of older adults.

## Conclusions

These data confirm a validated design for dietary assessment and includes usability results that can be used as references for comparison with future applications in this field. The raw data of the study (S2 Table) and the eNutri source code [30] were made publicly available. This work has potential contribution to promote wider uptake of online apps that can provide personalised nutrition advice at-scale, with potentially important implications for addressing pressing epidemiological challenges. General insights can also be applied in applications used in similar domains, especially in digital health.

## Supporting information

S1 Fig System Usability Scale (SUS) score for younger (n = 10 <60 years) and older (n = 10) adults in the formative study. (TIF)

S2 Fig FFQ completion time for younger (n = 10 <60 years) and older (n = 10) adults. (TIF)

S1 Table Formative study data sets. (XLSX)

S2 Table EatWellUK study data sets. (XLSX)
