# Supplementary figures and images for: Online dietary intake assessment using a graphical food frequency app (eNutri): Usability metrics from the EatWellUK study
Source: PLoS One. 2018 Aug 10;13(8):e0202006. doi: 10.1371/journal.pone.0202006 (PMC6086444; doi:10.1371/journal.pone.0202006)

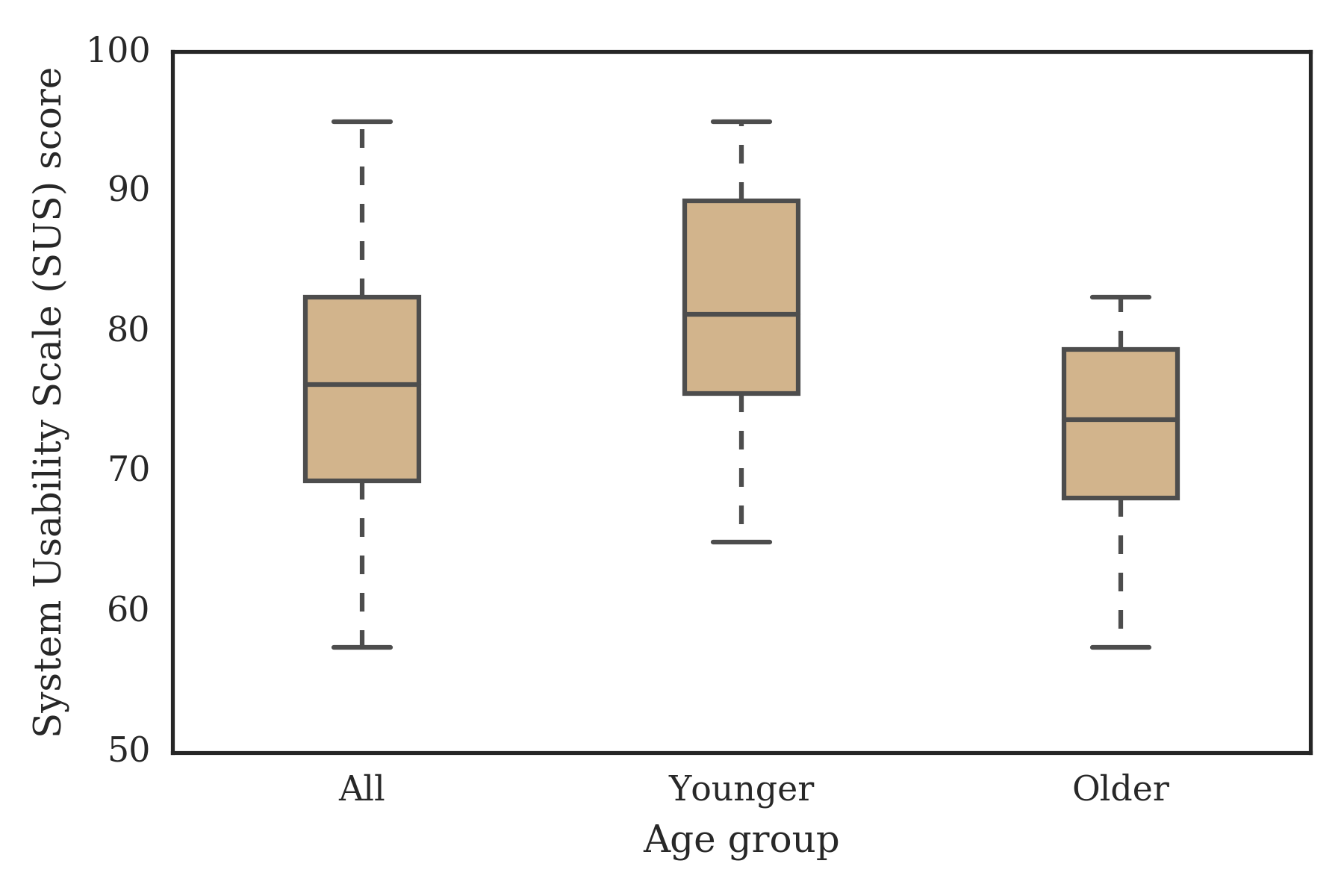

Supplement: S1 Fig — (TIF) [file pone.0202006.s001.tif]

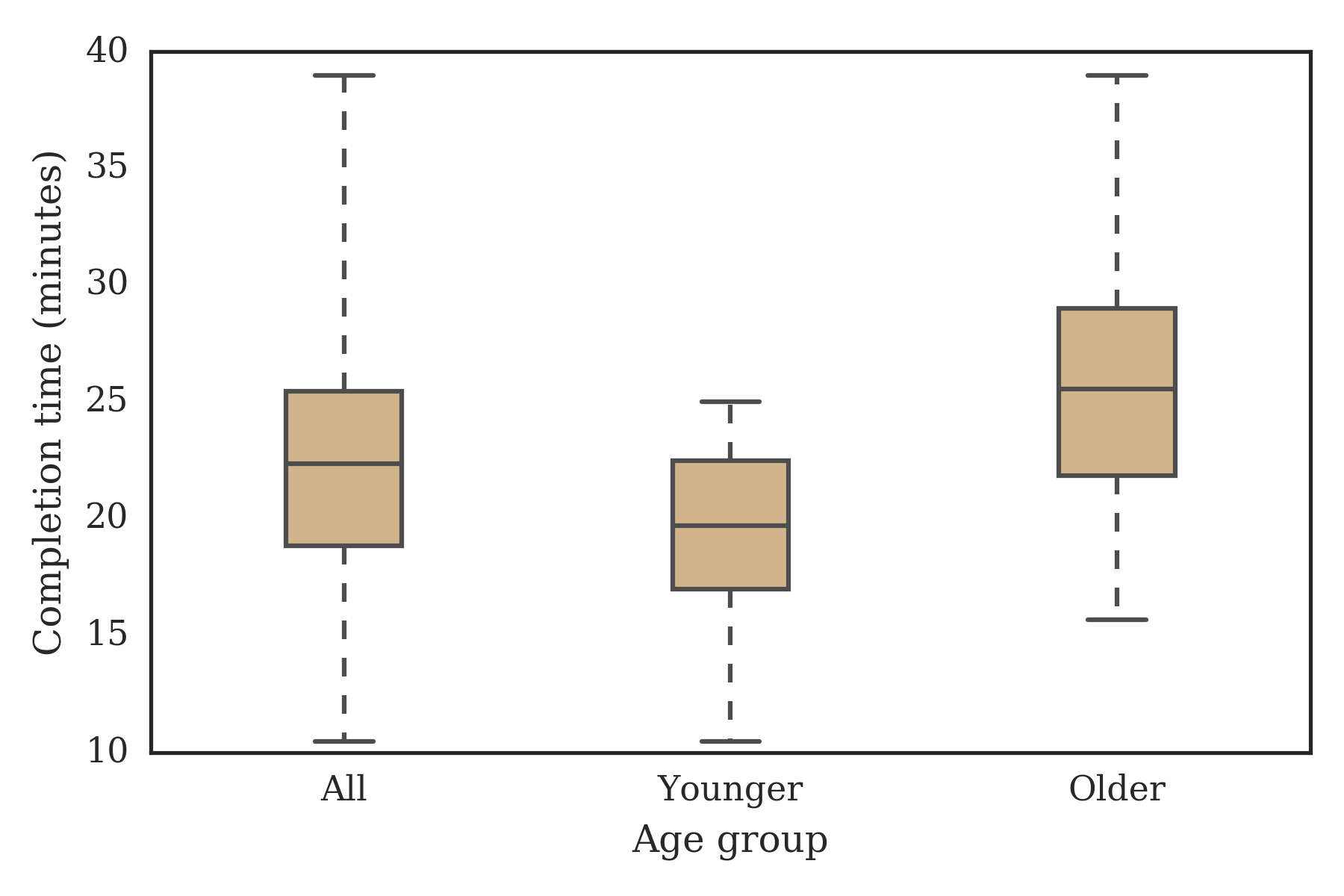

Supplement: S2 Fig — (TIF) [file pone.0202006.s002.tif]
